# The Role of Emotional Dysregulation, Impulsivity Traits and Aggressive Behaviors in Adolescents Who Sustain Multiple Motor-Vehicle Crashes

**DOI:** 10.3390/brainsci12121599

**Published:** 2022-11-22

**Authors:** Silvia Cimino, Paola Di Vito, Luca Cerniglia

**Affiliations:** 1 Department of Dynamic Clinical and Health Psychology, Sapienza University of Rome, Via degli Apuli, 1, 00186 Rome, Italy; 2 Faculty of Psychology, International Telematic University Uninettuno, Corso Vittorio Emanuele II, 39, 00186 Rome, Italy

**Keywords:** adolescence, motor vehicle accidents, emotional dysregulation, impulsivity, aggressive behaviors

## Abstract

Adolescents tend to experience multiple motor-vehicle crashes (MVCs). Recent literature has thoroughly explored the psychological consequences following an MVC, but prior psychological functioning, the ability to regulate one’s emotions and tendencies to impulsivity and aggressive behaviors have been far less explored. This study aims to explore the emotional–behavioral functioning, measured with the Youth Self-Report (YSR); emotional dysregulation, measured with the Difficulties in Emotional Regulation Scale (DERS); impulsivity traits, measured with the Barratt Impulsiveness Scale (BIS-11); and the capacity to be mindful, measured with the Mindful Attention Awareness Scale (MAAS), in adolescents that have experienced one or more MVCs in a year. N = 295 adolescents who visited an emergency department for MVCs were divided in two groups based on the number of MVCs they had sustained over the course of a year. The adolescents in Group 1 sustained more than three MVCs, and adolescents in Group 2 sustained only one. Participants were assessed through self-report questionnaires. Adolescents showed difficulties in emotional regulation, impulsivity traits, aggressive behaviors and a low capacity to be mindful. These results may be useful in the creation of intervention and prevention programs focused on emotional awareness targets for adolescents.

## 1. Introduction

Traffic accidents are a serious public-health problem. The World Health Organization points out that road crashes are one of the leading causes of death for young people and the subsequent development of disability to date worldwide [1]. In Italy, motor vehicle crashes (MVCs) are the primary cause of death in young people. In general, the risk of being involved in a collision is higher for adolescents between 16 and 19 years. Governments continuously implement strategies aimed at improving safety while driving, such as strict penalties, advertising campaigns on different media channels and drivers’ education. Although these strategies seem to have a positive effect and play an important role in accident prevention [2], motor vehicle crashes are still a worrying phenomenon. In Italy, the National Road Safety Plan (NSSP) 2030 [3], in addition to providing actions aimed at achieving the overall goal of halving the total number of deaths and serious injuries, identifies priority actions for improving the road safety conditions of certain categories of users particularly at risk, including early adolescents. However, the hoped-for positive effects are not yet tangible and the goal of “zero fatalities” is still a long way off. In fact, those injured in collisions are predominantly young people between the ages of 15 and 29 (30.3% of the total). For adolescents, traffic crashes increased more than 40 percent compared to 2020. In terms of gender, males are significantly more affected by traffic accidents than females [3]. Moreover, male adolescents tend to show higher levels of driver aggression, sensation seeking and general risk-taking compared to females of the same age [4]. Studies tend to explain this tendency with evidence that male adolescents seem to be generally more impulsive and tend to perceive their behaviors as less risky when compared to females of the same age [5,6].

### 1.1. Risk Factors for MVCs in Adolescence

Recent literature agrees on the identification of two broad categories of factors that seem to be associated with traffic accidents in youth. The first category includes factors that are related to traffic conditions while the second category includes all the “human” factors. This second category is considered to be the most influential [7]. To date, neurobiological and cognitive maturation models are considered the gold standard for understanding problem behaviors in adolescence, especially in the area of at-risk behaviors, such as driving [8]. However, in line with a multidisciplinary approach typical of the developmental psychopathology framework [9], considering this phenomenon as a result of the complex interplay of neurobiological, cognitive and individual psychological factors is of cardinal importance. When we are faced with young drivers returning to the emergency room several times, we are less prone to consider this fact just as the result of several and repeated distractions but as an acting out aimed at obtaining psychological help of some form or even as a way to regulate their emotions [10,11]. Adolescents find themselves in a developmental phase characterized by cognitive and neurobiological development and psychosocial challenges as well as continuous changes in emotional regulation mechanisms in stressful situations [12]. While changes in brain structure and function are a cardinal element sustaining these developmental paths in adolescence [13], developmental neuroimaging studies do not support a simple model of biological immaturity. In fact, the literature also underlines the importance of changes in social and affective processes, which begin around the onset of puberty, as crucial to understanding adolescents’ risk-taking tendencies [14]. It is a well-known fact that distraction may play a crucial part in traffic collisions [15]. The use of mobile phones when driving, for example, is considered one of the most distracting factors [16]. Driver distraction lowers risk perception [17]. On this basis, a vast body of literature underlines how adolescents—who are more prone to fail to identify dangerous situations—are more likely to experience MVCs [18,19]. The recent literature [20] has underlined that mindfulness practices may be particularly of help in reducing the possibility of traffic collisions. The construct of mindfulness involves the ability to self-regulate attention to keep it focused on immediate experience, thereby allowing for increased recognition of mental and material events in the present moment [21]. It is true that risk-taking exponentially increases during adolescence due to the brain’s socioemotional remodeling of the dopaminergic system, leading to increased striving for reward-seeking situations, especially when peers are present [22]. In a developmental psychopathology framework [9], adolescents often recur to risky behaviors because of a developmental striving to gain independence from their parents and gain authority in the eyes of their peers [23]. Moreover, it is important to underline that while adolescents’ cognitive abilities reach adult levels at the age of 16, psychosocial maturity is a whole other challenge reaching maturity level around or beyond the age of 18 [24]. This creates a maturity gap between cognitive and psychosocial development [24]. The contribution that our perspective hopes to make is that while previous empirical studies have often indicated distraction and attention problems among the factors most frequently associated with traffic accidents in adolescence [25], we argue that, notwithstanding the relevance of these findings, the possible distraction at the time of the accident could be an important indicator of emotional and behavioral dysregulation and not merely due to contextual cognitive failures.

### 1.2. A Developmental Psychopathology Approach to MVCs

Notwithstanding the relevance of these factors, studies confirm that traffic collisions, especially in adolescence, are a complex phenomenon and that the personal and psychological characteristics of these young drivers should be taken into serious consideration [26]. One particularly interesting factor that has been covered in recent studies is impulsivity [27,28]. Impulsivity has been identified as a peculiar trait for many at-risk behaviors in adolescence, such as binge eating disorders and MVCs [29,30]. The recent literature confirms that impulsive adolescents tend to operate affective (rather than cognitive) prompting in the context of decision making when driving, making them particularly vulnerable to the risk of accidents. Although there is a vast literature that takes into consideration the effects of an MVC on a psychological level, such as depression [31,32], anxiety [33] and PTSD-like symptoms [34], far fewer studies have addressed the emotional functioning of adolescents before the collision [26,35,36]. These variables are often considered as outcomes of the MVC, but they could also be investigated as predictors. For instance, a particular type of emotional–behavioral functioning could make an adolescent more vulnerable to the possibility of an MVC. Emotional dysregulation, for example, appears to be an important transdiagnostic factor that increases the risk for a wide range of psychopathology outcomes in adolescence, including risk behaviors [37]. On the contrary, emotional intelligence—that is the ability and capacity to be aware of, control and express one’s emotions—has been argued as needing to be included in driving education and traffic safety programs [38]. Emotional dysregulation is also associated with aggressive behavior in adolescence [39]. Although it is true that around half of adolescents experience aggressive symptoms that impair their psychological functioning at some point in this phase [36,40], we should not underestimate the possible maladaptive role of aggressive behaviors, especially as they are often associated with perilous situations [41,42]. To date, no studies have investigated the possible role of aggressive behaviors as a predictor of the possibility of being involved in an MVC in adolescence. However, the literature has repeatedly underlined that aggressive behaviors in adolescence may be a way to express one’s feelings, especially when the adolescent has not learnt the ability to communicate, express and regulate his emotions [43]. Taking these studies into consideration, and on the basis of the previous literature [26,38,44,45], we aimed to investigate two groups of adolescents to underline the associations between their psychological functioning and the risk of MVCs: one group who sought help at the emergency room for an MVC only once over the course of one year (Group 2; N = 154), and one group who sought help more than three times in a year after being involved in an MVC (Group 1; N = 141). We aimed to investigate the adolescents’ general emotional–behavioral functioning, emotional dysregulation difficulties, impulsivity traits and the ability to be mindful. Moreover, we wanted to further investigate the possible contribution of these psychological variables on the possibility of being involved in an MVC. We hypothesized that:

- Adolescents who sustained multiple MVCs over the course of one year (Group 1) would show more problematic emotional–behavioral functioning;
- Adolescents who sustained multiple MVCs over the course of one year (Group 1) would show higher levels of emotional dysregulation and impulsivity traits and a lower ability to be mindful;
- Emotional dysregulation, impulsivity traits and aggressive behaviors would predict the likelihood of being involved in multiple MVCs.

## 2. Materials and Methods

We used a consecutive sampling method to create a convenience sample. We followed the groups’ division adopted in previous studies on the same topic [31,39] on the basis of the recommendations in Marcelli’s studies [46] on motor-vehicle collision recidivism in adolescence. The inclusion criteria are presented here: (1) no psychiatric diagnosis in the adolescents; (2) no presence of PTSD-like symptoms or acute stress at the moment of recruitment as assessed with the SCID I (Non-Patient Edition) [47]; and (3) no other current medical and/or psychological treatment. We recruited 295 adolescents (N = 295; age range: 13–16 years; mean age: 14.57, SD: 0.98) who visited an Italian emergency department after an MVC. In the text, we refer to these accidents as MVCs, but it is necessary to emphasize that they included mopeds and scooters with an engine capacity of 125 cubic centimeters and electric bikes.

We excluded from the sample all the adolescents who were passengers at the moment of the collision; adolescents who suffered serious injuries; adolescents who did not give consent to participate in the study; adolescents whose parents did not give consent to take part in our study; adolescents who had a psychiatric diagnosis. For serious head injuries, in collaboration with the nursing staff, we excluded from the sample subjects who showed the following symptoms at the time of the accident: loss of consciousness, disabling headaches, convulsions or seizures, or repeated vomiting. For those who sustained multiple MVCs, we asked, while collecting anamnestic information, whether they had sustained serious brain damage in previous incidents. We excluded those adolescents too.

Most of the adolescents were Caucasian (94.1%) and 71% of their families had a household income of between EUR 28.000 and 55.000 per year. Of the adolescents, 82.2% were from intact family groups and 72% were the firstborn. In line with the Declaration of Helsinki, the Ethical Committee of La Sapienza, University of Rome authorized the research plan before the start of the study. All the participants were asked to complete an informed consent document. The anonymity and privacy of any personal information was guaranteed. The participants completed the following self-report tools.

The Youth Self-Report/11-18 (YSR/11-18) [48] is a 112-item self-report questionnaire targeted for young people that investigates behavioral and emotional difficulties. The tool is an empirically validated measure that showed internal consistency in the Italian context [49]. Each item is scored on a 3-point Likert scale where 0 is not true, 1 is somewhat or sometimes true and 2 is very or often true. This tool allowed the following subscales to be derived: Withdrawn, Somatic complaints, Anxious/Depressed, Social problems, Thought problems, Attention problems, Delinquent behavior, Aggressive behavior, and Self-destruction identity. The tool has good internal consistency with Cronbach’s alphas ranging from 0.71 to 0.95.

The Mindful Attention Awareness Scale (MAAS) [21] is a 15-item self-report scale designed to assess characteristics of dispositional mindfulness, that is, receptive awareness of and attention to what is taking place in the actual moment. This tool is proven to show strong psychometric properties and has been validated with college, community and clinical patient samples. The measure takes around 10 min to complete making it particularly useful for research purposes. The Italian version of the MAAS shows good psychometric properties [50].

The Difficulties in Emotional Regulation Scale (DERS) [51] is a 36-item self-report scale that measures emotional regulation problems by asking participants how they relate to their emotions. The tool is built on an integrative conceptualization of the construct of emotional regulation involving not only arousal modulation but also awareness, understanding, the ability to accept emotions and the ability to control one’s behavior regardless of current emotional state. The tool gives the possibility of deriving the following subscales: Non-acceptance of emotional responses; Difficulty engaging in goal-directed behavior; Impulse control difficulties; Lack of emotional awareness; Limited access to emotional regulation strategies; Lack of emotional clarity. The tool has been validated in the Italian context showing good internal consistency and adequate test-retest reliability [52].

The Barratt Impulsiveness Scale (BIS-11) [53] is a 30-item self-report questionnaire designed to evaluate different subtypes of impulsivity. Items are scored on a 4-point Likert scale: Rarely/Never = 1; Occasionally = 2; Often = 3; Almost Always/Always = 4. The Italian validation of the BIS-11 shows good psychometric properties [54].

## 3. Results

### 3.1. Data Analysis

We carried out a series of multivariate analyses of variance (MANOVAs) in Group 1 and Group 2 to assess the adolescents’ psychological functioning and possible differences in emotional dysregulation, impulsivity traits and the ability to be mindful. Subsequently, we conducted a stepwise regression to examine the possible role of these variables in predicting the likelihood of being involved in an MVC. All analyses were performed with SPSS software (Version 27).

#### 3.1.1. Differences in Emotional–Behavioral Functioning

Means and standard deviations for emotional–behavioral functioning in Groups 1 and 2 are shown in Table 1.

Results of the MANOVA confirmed that there was a statistically significant difference between the two groups in the following combined dependent variables: Withdrawn (F = 28.03; *p* < 0.001); Social problems (F = 13.66; *p* < 0.001); Thought problems (F = 25.19; *p* < 0.001); Attention problems (F = 65.34; *p* < 0.001); Delinquent behavior (F = 29.78; *p* < 0.001); Aggressive behavior (F = 46.51; *p* < 0.001); and Self-destructive identity (F = 13.95; *p* < 0.001).

Based on these results, adolescents in Group 1 and Group 2 significantly differed in their emotional–behavioral functioning. Moreover, 98.04% of Group 1 surpassed the clinical cut-off score for the YSR total while only 34.01% of Group 2 showed such scores. Male and female adolescents did not show any significant difference in any of the considered variables.

#### 3.1.2. Differences in Emotional Dysregulation, Impulsivity Traits and Mindfulness

Means and standard deviations for Difficulties in Emotional Regulation, Impulsivity traits and Mindfulness in Groups 1 and 2 are shown in Table 2.

Results of the MANOVA confirmed that there was a statistically significant difference between the two groups in the following combined dependent variables: Difficulties in Emotional Regulation (F = 589.75; *p* < 0.001); Impulsivity (F = 125.12; *p* < 0.001); and Mindfulness (F = 600.65; *p* < 0.001).

Based on these results, adolescents in Group 1 and Group 2 significantly differed in the aforementioned variables.

#### 3.1.3. The Contribution of Psychological Functioning to the Likelihood of Being Involved in Multiple MVCs

Based on the rather sparse literature on the subject, and therefore not having any studies that investigated the same variables, we decided to use a stepwise regression model to derive a more accurate statistical model based on changes in R-square. A stepwise linear regression was used to explore the influence of potential predictors on the number of MVCs out of the socio-demographic variables (age and gender), the YSR subscales, the DERS scale, the BIS scale and the MAAS scale. At each step, variables were chosen based on *p*-values, and the *p*-value threshold of 0.001 was used to set a limit on the total number of variables included in the final model. The following variables showed a high association with the number of incidents in that the higher the scores on Emotional Dysregulation (R2 = 0.67; β = 0.49; *p*  < 0.001), Impulsivity (R2 change  =  0.13; β  = 0.11; *p*  < 0.001), Self-destructive behavior (R2 change  =  0.01; β  = 0.12; *p*  < 0.001) and Aggressive behavior (R2 change  =  0.01; β  = 0.10; *p*  <  0.001), the more likely individuals were to have experienced multiple MVCs (F  = 207.29 , *p*  <  0.001). The final regression model explained 82% of MVC variance (*p*  <  0.001). Age and gender were excluded from the model as they did not contribute to its explanation.

## 4. Discussion

The purpose of the present study was to investigate the emotional–behavioral functioning (aggressive behaviors in particular), emotional dysregulation, impulsivity traits and the ability to be mindful in adolescents who experienced one or more than three motor vehicle collisions (MVCs) in one year. Further, we intended to verify the possible contribution of these variables to the likelihood of being involved in several MVCs. Regarding our first aim, in line with other studies [26,31], we expected adolescents in Group 1 who sustained more than three MVCs over the course of one year to show more problematic emotional–behavioral functioning. This hypothesis was confirmed by our analysis. Consistent with other studies [44,55], Group 1 showed higher levels of both internalizing and externalizing problems, namely aggressive, delinquent and self-destructive behaviors. Aggressive symptoms, the inability to regulate one’s emotions and a tendency toward impulsivity were found to explain, in our model, adolescents’ likelihood of being involved in MVCs on a recurrent basis. Our second hypothesis was confirmed as well. We expected adolescents who sustained multiple MVCs to show higher levels of emotional dysregulation and impulsivity traits and a lower ability to be mindful, and this was supported by our data. As underlined by other literature [35], this behavioral dysregulation, due both to neurobiological immaturity and to psychological factors, could play an important role in adolescents’ risk taking. What is particularly interesting to point out is that the variables that help explain this link are not cognitive variables but refer to an area of emotional regulation—or rather dysregulation—that seems to be particularly interesting when taking this phenomenon into consideration. Attention problems, thought problems and the capacity to be mindful did not seem to influence our predictive model. For instance, MVC recidivism of collisions among adolescents is quite common. It is estimated that one in four teenagers will have a relapse within the year following their first accident [7]. We also included adolescents’ gender in our model. However, we did not find a significant effect of gender in adolescence. The recent literature [26,56] takes some divergent positions on the role of gender in traffic accidents and risk perception. Some studies [56,57] emphasize that risk perception regarding road safety seems to be equal in males and females, but males seem to be less concerned about the risk of a traffic accidents than females.

### 4.1. An Explanatory Model of MVC Recidivism in Adolescence

MVC recidivism among adolescents can be considered a form of acting out caused by psychological difficulties, such as the inability to identify and express their own emotions [58]. While it is possible and quite common to have an MVC during adolescence, recidivism and therefore returning to emergency departments more than three times per year is a worrying scenario that should be further investigated in order to build more effective prevention programs. MVCs and traffic accidents in general may pose a serious challenge to adolescents for their physical and psychological well-being after the collision as they have been proven to increase the risk for subsequent psychological problems. Being involved in an MVC seems to be a predictor for developing psychopathology, and untreated psychological consequences could have long-term effects on the adolescents’ mental health [59]. The most common outcomes of MVCs are usually PTSD-like symptoms, anxiety, depression, aggressive and self-aggressive behaviors, phobias and emotional dysregulation in general [60]. Notwithstanding the importance of this finding, the strength of our study is to consider these variables as important “before factors” that can play an important part in predicting adolescents’ risk behaviors related to the possibility of being involved in MVCs, especially for what concerns emotional dysregulation and aggressive behaviors. We speculate that accidents involving adolescents who recidivate in this behavior cannot be attributed solely to inexperience or ignorance—whether deliberate or not—of safety practices but that this behavior stems from a complex interweaving of variables that have to do with deep underlying psychological suffering, which may take the form of impulsivity or driving aggression but that refers to an underlying difficulty in regulating emotions.

### 4.2. The Importance of Psychological Screening and Prevention Programs

Notwithstanding the limitations we mention in the next paragraph, these findings in our study may be useful for mental health professionals when planning prevention and intervention programs for adolescents. Giving adolescents the opportunity to focus on their ability to identify, express and regulate their emotions in order to decrease risky and impulsive behaviors could potentially be beneficial in reducing the phenomenon of multiple MVCs in young people. Emergency rooms could integrate psychological screening processes for adolescents who sustain an MVC in order to give them the possibility of reaching out to local and public services that offer psychological help.

### 4.3. Limitations

Our study has some limitations. On one hand, the homogeneity of ethnicity and socioeconomic status of our sample do not enable broad generalizations of our results. On the other hand, we only used self-report questionnaires which could impair the accuracy of the constructs we measured. Moreover, we did not assess the quality of peers’ relationships and how this can be an important risk or protective factor associated with risky behaviors in adolescence as much as the other variables we considered can be [61]. Several studies suggest that when adolescents drive with peer passengers, they are more likely to perform an aggressive act, such as an illegal maneuver [26,62], increasing the risk of a collision. Peer recognition is a fundamental factor in adolescence that can lead young people to indulge in risky behaviors and practices to impress their peers [63], and that needs to be further investigated in order to explain the complex relations behind multiple MVCs during adolescence. Furthermore, head injuries after an MVC can occur in multiple locations. Although we tried to exclude subjects who declared a head injury after the accident, the uncontrolled source of variance of the differing locations of head injuries potentially sustained after the accident and their effect on the psychological functioning of these adolescents is a limitation of our study. Another limitation we want to address is the lack of a control group in our study as all the adolescents (Groups 1 and 2) sustained at least one MVC.

## Figures and Tables

**Table 1:** Mean scores, standard deviations for Groups 1 and 2 on the YSR scales.

YSR Scale	Group 1 MEAN (SD)	Group 2 MEAN (SD)	η^2^	*p*
Withdrawn	10.68 (2.33)	9.18 (2.51)	0.12	<0.001
Somatic complaints	9.58 (2.99)	9.14 (3.01)	0.08	<0.056
Anxious/depressed	17.10 (4.01)	17.15 (5.33)	0.02	<0.077
Social problems	8.26 (2.04)	7.44 (1.74)	0.45	<0.001
Thought problems	6.64 (1.75)	5.58 (1.86)	0.03	<0.001
Attention problems	8.46 (2.23)	6.40 (2.12)	0.06	<0.001
Delinquent behavior	5.55 (1.09)	4.9 (0.76)	0.34	<0.001
Aggressive behavior	19.62 (4.44)	16.05 (4.54)	0.67	<0.001
Self-destructive identity	11.87 (2.67)	10.74 (2.52)	0.56	<0.001

**Table 2:** Mean scores, standard deviations for Groups 1 and 2 on the DERS, BIS-11 and MAAS scales.

	Group 1 MEAN (SD)	Group 2 MEAN (SD)	η^2^	*p*
Difficulties in Emotional Regulation	82.75 (12.55)	55.38 (5.89)	66.75	<0.001
Impulsivity	78.57 (8.16)	68.33 (7.53)	43.12	<0.001
Mindfulness	3.15 (0.35)	4.03 (0.26)	68.65	<0.001

## Data Availability

Data are available on request to the corresponding author.
